# Place of Death Before and During the COVID-19 Pandemic

**DOI:** 10.1001/jamanetworkopen.2023.50821

**Published:** 2024-01-08

**Authors:** Ben Teasdale, Aditya Narayan, Stephanie Harman, Kevin A. Schulman

**Affiliations:** 1 School of Medicine, Stanford University, Stanford, California; 2 Clinical Excellence Research Center, Stanford University, Palo Alto, California; 3 Department of Medicine, Stanford University, Stanford, California; 4 Graduate School of Business, Stanford University, Palo Alto, California

## Abstract

This quality improvement study examines the national and ongoing impact of the COVID-19 pandemic with the place of death among individuals in the US.

## Introduction

As recently as 2015, dying at home became more common than dying in a hospital.^1^ The COVID-19 pandemic interrupted these trends, as the acute clinical course of severe infection shifted deaths back inside hospitals. Beyond the direct consequence of pandemic-related mortality, indirect associations of factors, such as workforce and resource limitations, disrupted the provision of end-of-life care more broadly, even for patients who were not directly infected with COVID-19.^2^ Three years after the start of the pandemic, we investigate the national and ongoing impact of the COVID-19 pandemic and place of death among individuals in the US.

## Methods

This study followed the Strengthening the Reporting of Observational Studies in Epidemiology (STROBE) reporting guideline and was deemed exempt by the institutional review board of Stanford University. Informed consent was waived because data were publicly available and deidentified.

The nationally representative US Centers for Disease Control and Prevention (CDC) Wide-ranging Online Data for Epidemiologic Research (WONDER) database was queried for mortality and place of death data.^3^ Time trends of mortality data were reported from January 2010 to June 2023 as mean daily death rates, separating COVID-19 deaths (*International Statistical Classification of Diseases and Related Health Problems, Tenth Revision *[*ICD-10*] U07.1) from all-cause mortality. Place of death was recorded using prespecified variable definitions within CDC WONDER, including hospital, home, nursing facility, hospice facility, and other (eg, unknown place of death).

Interrupted time-series analyses were performed to assess how trends in place of death changed after the onset of the pandemic.^4^ The pandemic was quantified as relative risk (RR), comparing the risk of death in a specific location before and after the onset of the COVID-19 pandemic. To account for underlying time trends, segmented OLS linear regressions were constructed, with the pre–COVID-19 model constructed using data from March 2017 to March 2020 and the post–COVID-19 model constructed using data from March 2020 to March 2023. To approximate both the immediate and ongoing disruption, RRs were calculated on March 2020 and March 2023. All statistical tests were 2-sided at a significance level of *P* < .05 and were completed using Stata version 18.0 (StataCorp) in October 2023.

## Results

This quality improvement study examined 38 300 000 deaths from January 2010 to June 2023 (18 700 000 [48.7%] female; 19 600 000 [51.3%] male; 21 200 000 [73.4%] age older than 65 years; 4 700 000 [12.2%] Black individuals; 32 300 000 [84.4%] White individuals), including 1 000 000 deaths attributable to COVID-19 (Figure and Table). At the onset of the COVID-19 pandemic (ie, March 2020), there was an 11% increase in in-hospital mortality (RR, 1.11; 95% CI, 1.05-1.17), with non–COVID-19 in-hospital mortality decreasing by 13% (RR, 0.87; 95% CI, 0.82-0.93). There was a 19% increase in non–COVID-19 home deaths (RR, 1.19; 95% CI, 1.13-1.26), a 13% decrease in non–COVID-19 deaths in nursing facilities (RR, 0.87; 95% CI, 0.81-0.94), and a 22% decrease in non–COVID-19 deaths within hospice facilities (RR, 0.78; 95%, CI, 0.70-0.88).

**Figure.**
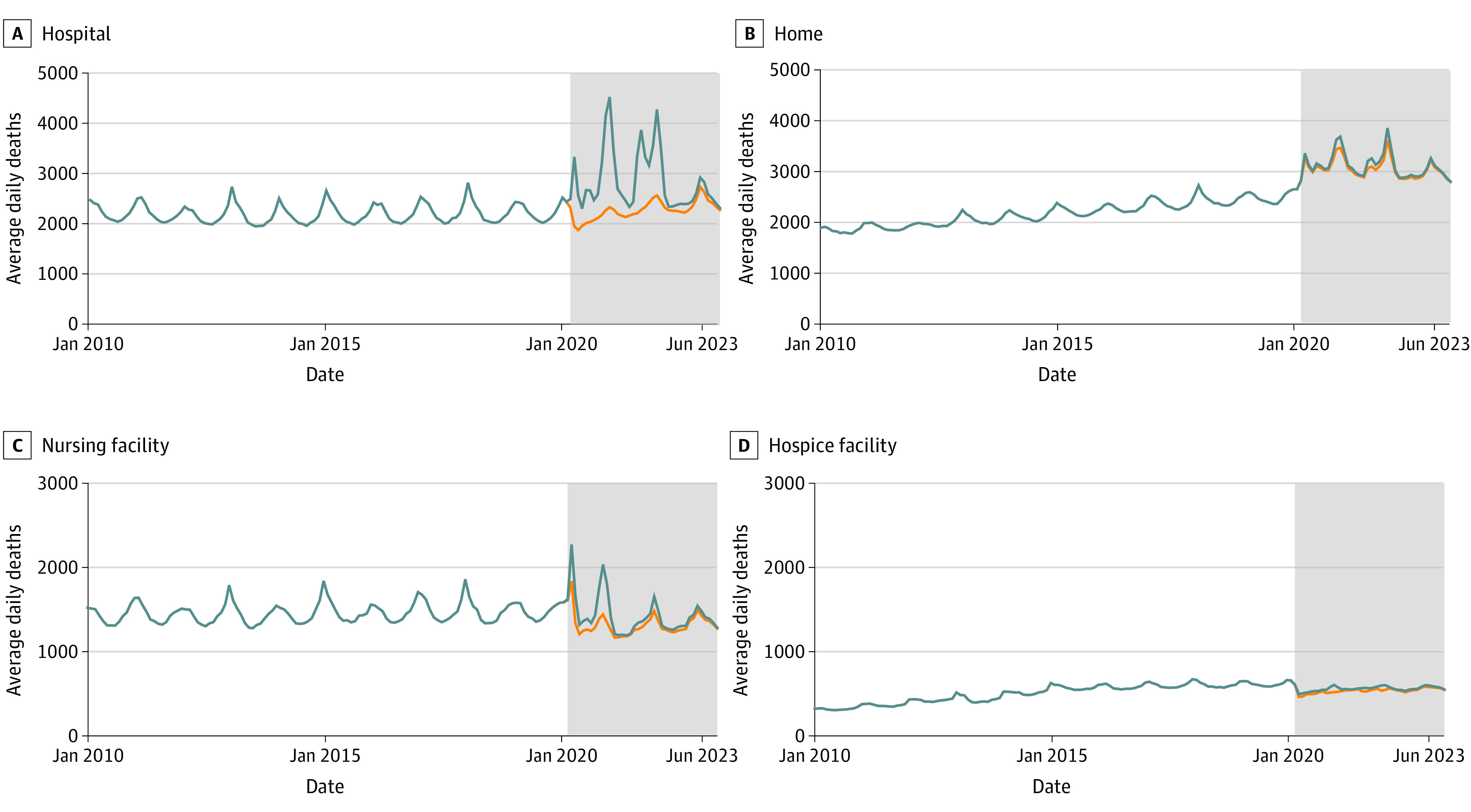
Trends in Place of Death Before and During the COVID-19 Pandemic Data regarding place and cause of death obtained from National Health Statistics Mortality Data on the US Centers for Disease Control and Prevention (CDC) Wide-ranging Online Data for Epidemiologic Research (WONDER) database, including January 2005 through June 2023.^3^ The CDC WONDER database reports deaths as monthly totals, which are reported here as average daily deaths. Cause of death is differentiated as non–COVID-19 (orange) or all-cause mortality (blue). The COVID-19 pandemic is shown (gray), starting in March 2020.

**Table.** Association of COVID-19 Pandemic With Place of Death^a^

Outcome	Average daily deaths (%)		Relative risk (95% CI)	
	Pre–COVID-19	Post–COVID-19	March 2020	March 2023
**Hospital**				
Total	2263 (32.8)	3048 (36.4)	1.11 (1.05-1.17)	1.11 (1.05-1.17)
Non–COVID-19	NA	2049 (29.2)	0.87 (0.82-0.93)	1.05 (0.99-1.11)
**Home**				
Total	2541 (36.8)	3219 (38.5)	1.04 (0.99-1.10)	1.04 (0.98-1.09)
Non–COVID-19	NA	3134 (44.6)	1.19 (1.13-1.26)	1.06 (1.00-1.11)
**Nursing facility**				
Total	1482 (21.4)	1564 (18.7)	0.87 (0.81-0.93)	0.80 (0.74-0.86)
Non–COVID-19	NA	1335 (19.0)	0.87 (0.81-0.94)	0.83 (0.77-0.90)
**Hospice facility**				
Total	623 (9.0)	541 (6.5)	0.71 (0.64-0.80)	0.83 (0.75-0.93)
Non–COVID-19	NA	506 (7.2)	0.78 (0.70-0.88)	0.84 (0.75-0.94)

Abbreviation: NA, not applicable.

^a^ Data regarding place and cause of death were obtained from the National Health Statistics Mortality Data on US Centers for Disease Control and Prevention Wide-ranging Online Data for Epidemiologic Research database.^3^ Outcomes were calculated using an interrupted time series regression model using data from March 2017 through March 2023, segmenting the models in March 2020. Relative risks are calculated comparing location-specific deaths as a portion of total deaths, comparing rates calculated using the post–COVID-19 model with the pre–COVID-19 model. Relative risk is calculated in March 2020 as an approximate measure of the immediate impact of the pandemic and March 2023 as an approximate measure of the ongoing impact of COVID-19.

Three years after the start of the pandemic (ie, March 2023), increases in in-hospital mortality remain when compared with projections made using the pre–COVID-19 model (RR, 1.11; 95% CI, 1.05-1.17), while in-hospital mortality for non–COVID-19 diagnoses are nonsignificantly increased (RR, 1.05; 95% CI, 0.99-1.11). Non–COVID-19 home death rates remain significantly elevated compared with prepandemic trends (RR, 1.06; 95% CI, 1.00-1.11), while non–COVID-19 death rates within nursing and hospice facilities remain significantly decreased (nursing facility: RR, 0.83; 95% CI, 0.77-0.90; hospice facility: RR, 0.84; 95% CI, 0.75-0.94).

## Discussion

Prior studies showed how COVID-19 exacerbated access issues at the end of life immediately after the onset of the pandemic. For example, Medicaid patients from Washington were more likely to die in a hospital or without hospice services.^5^ Our study finds that pandemic-related disruptions to place of death trends are ongoing, national, and extend to non–COVID-19–related diagnoses. Our study is limited by the imperfect classification of the place of death variables within the CDC WONDER database, including ambiguity regarding how assisted living facilities should be classified. Furthermore, changes in hospice facility use reflect only a small portion of hospice care overall.

While the pandemic strained end-of-life services, it also coincided with preexisting concerns, such as issues with hospice quality measures and regulation of for-profit hospices acquired by private equity firms.^6^ Our study provides further evidence that additional focus to ensure access to end-of-life services is necessary.

## Notes

Supplement.
Data Sharing Statement
