# Supplementary material for: Place of Death Before and During the COVID-19 Pandemic
Source: JAMA Netw Open. 2024 Jan 8;7(1):e2350821. doi: 10.1001/jamanetworkopen.2023.50821 (PMC10774989; doi:10.1001/jamanetworkopen.2023.50821)
Supplement: Supplement. — Data Sharing Statement [file jamanetwopen-e2350821-s001.pdf]

Data Sharing Statement

Teasdale. Place of Death Before and During the COVID-19 Pandemic. *JAMA Netw Open*.
Published January 08, 2024. doi:10.1001/jamanetworkopen.2023.50821

Data
Data available: No

Additional Information
Explanation for why data not available: Data used in this study was obtained from a already publicly available database maintained by the CDC (WONDER).
